# Pneumoperitoneum After Jejunostomy Tube Placement Managed by Needle Decompression: A Case Report

**DOI:** 10.7759/cureus.44027

**Published:** 2023-08-24

**Authors:** Khalid Al Shamousi, Masoud Salim Kashoob, Jawahir Lal, Said A Al-Busafi

**Affiliations:** 1 Gastroenterology Unit, Department of Medicine, Sultan Qaboos University Hospital and College of Medicine and Health Sciences, Sultan Qaboos University, Muscat, OMN; 2 Internal Medicine Training Program, Oman Medical Specialty Board, Muscat, OMN; 3 Gastroenterology Unit, Department of Medicine, Sultan Qaboos University Hospital, Sultan Qaboos University, Muscat, OMN

**Keywords:** enteric nutritional support, feeding tube placement, needle decompression, pneumoperitoneum, percutaneous endoscopic jejunostomy

## Abstract

Percutaneous endoscopic feeding tube placement is a commonly performed procedure in patients who cannot take food by mouth. While it is considered a safe and effective method of providing nutritional support, like any medical procedure, it can lead to complications. Feeding tube placement, including percutaneous endoscopic jejunostomy (PEJ), is associated with several complications, including bleeding, site infection, aspiration, buried bumper, tube dislodgement, and pneumoperitoneum. We report a case of a 20-year-old male with multiple medical issues who underwent a PEJ that was complicated by bowel distension. The patient developed tension pneumoperitoneum post-procedure, which was treated with a bedside needle decompression. This case report highlights the significance of promptly recognizing and intervening in complications that may arise during a frequently performed medical procedure, PEJ tube placement, to prevent serious consequences, including bowel ischemia.

## Introduction

Percutaneous endoscopic tube placement is a routine procedure to support patients who are unable to tolerate adequate oral intake [1]. Even though it is a safe and effective method allowing enteric nutritional support, it is associated with certain complications like any other procedure [2]. Percutaneous endoscopic jejunostomy (PEJ) is mainly used in cases of gastric pathology and gastric surgeries.

Some complications of PEJ tube placement include bleeding, PEJ site infection, aspiration, buried bumper, tube dislodgement, and pneumoperitoneum [3,4]. Pneumoperitoneum has been reported with varying frequencies (5-16%) in the literature, depending on the patient population and study period duration [3,5]. Pneumoperitoneum is typically self-resolving within 72 hours through the resorption of air [3,4]. However, in some cases, it might persist for a longer period. Despite being benign in most cases, there have been reports of major consequences, including death [6]. Hence, some interventionists might not feel comfortable ignoring the potential for these adverse consequences. We discuss a case of a patient with significant pneumoperitoneum that was managed by paracentesis with air evacuation during a PEJ tube placement procedure, a previously unreported intervention in this clinical setting.

## Case presentation

The patient was a 20-year-old male with a medical history of Fanconi anemia post bone marrow transplant for 10 years. He also had a history of moderately differentiated squamous cell carcinoma of the esophagus, invasive poorly differentiated squamous cell carcinoma of the tongue, and had undergone esophagectomy four years ago complicated by anastomotic stricture requiring esophageal stricture dilatation. He also had small bowel obstruction due to abdominal adhesions, which was managed conservatively. The patient was referred to the gastroenterology team for PEJ. After discussing the risks and benefits of the procedure, an upper GI endoscopy was performed, which showed a benign esophageal stricture between 14-25 cm from the incisors. A small needle with methylene blue was advanced under negative suction. Once the air was felt in the syringe, methylene blue was injected and seen in the distal duodenum/proximal jejunum, confirming direct access. However, due to the unavailability of CO_2_ for bowel inflation and since only air was available, the procedure was terminated due to bowel distension. Instead, the NGT was placed over the guide wire to treat in-line small bowel obstruction, and fluoroscopic confirmation was done before the procedure was terminated.

**Figure 1:**
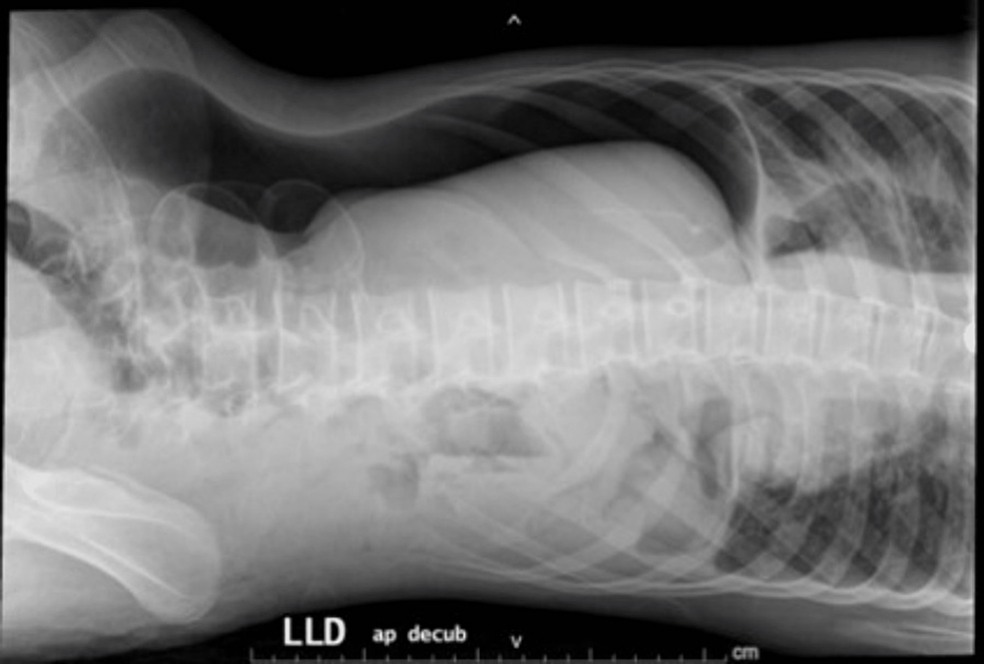
Left lateral decubitus abdominal X-ray The image demonstrates a crescent-shaped radiolucent area along the right side of the abdomen in keeping with the massive pneumoperitoneum. There are no dilated bowel loops

**Figure 2:**
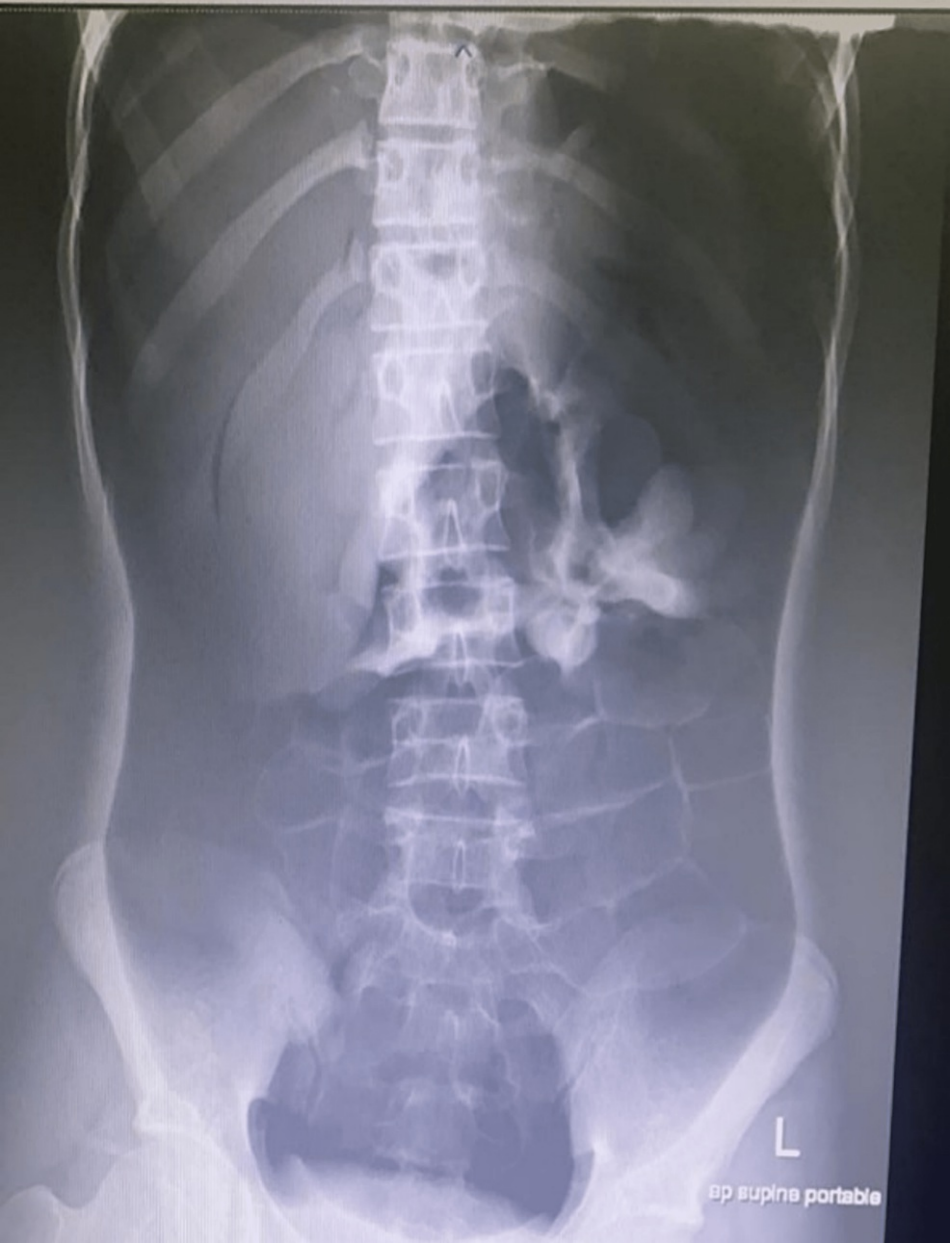
Supine portable abdominal X-ray The image demonstrates the pneumoperitoneum

Post-procedure, the patient developed abdominal distension and became tachycardic. An abdominal X-ray was done, which showed tension pneumoperitoneum, as shown in Figures 1-2.

After obtaining verbal consent, the patient was positioned supine, and the abdomen was sterilized using chlorhexidine. A 14 French cannula, connected to a water-filled syringe without a plunger through a three-way lock, was inserted into the upper area near the periumbilical region of the abdomen (Video 1). This was done until the air rushed out, indicating successful placement. Additional air was expelled by compressing the abdomen and instructing the patient to cough. To ensure safety, the needle was partially withdrawn into its sheath after the release of air.

**Video 1:** Bedside needle decompression of pneumoperitoneum using a 22 gauge needle attached to a water-filled syringe inserted through the periumbilical area

## Discussion

The presence of a small amount of air in the peritoneal cavity secondary to air inflated into the gastric lumen during endoscopy was always assumed to be a benign finding [1]. However, in a recent retrospective study of patients who underwent PEG tube placement, out of nine patients with proven pneumoperitoneum, five (55.5%) had clinically significant symptoms, including fever, abdominal tenderness, and leukocytosis [6]. Moreover, two of those five patients died from septic shock [6].

The mechanisms behind pneumoperitoneum are likely related to (i) elevated intragastric air pressures from the endoscope, (ii) air escape from the stomach after needle puncture [4,5], (iii) incomplete fixation of the PEG tube against the abdominal wall, and (iv) direct needle puncture into the abdominal cavity. In our case, it is difficult to pinpoint which of the above mechanisms was the exact cause, and instead, we assume that all of them were somehow contributory. 

Pneumoperitoneum alone can indicate a more concerning complication like intestinal perforation. Our patient’s pre-procedure clinical, laboratory, and radiological evaluation revealed no possible etiology or risk factor for pneumoperitoneum. Due to concerns for compressive symptoms post-procedure and the need for later decompression, as reported in several case reports [3,4,7], the decision was made to proceed with air evacuation by needle decompression guided by plain X-rays, resulting in the resolution of the pneumoperitoneum. The post-procedure course was uneventful, and the patient was subsequently discharged home.

## Conclusions

Pneumoperitoneum is a rare consequence of a commonly performed procedure, i.e., percutaneous endoscopic feeding tube placement; it can lead to severe consequences, including tension pneumoperitoneum with concomitant hemodynamic instability. Early detection of this adverse event is critical, and bedside needle decompression is a safe and readily available intervention to prevent major complications later.
